# High RIG-I and EFTUD2 expression predicts poor survival in endometrial cancer

**DOI:** 10.1007/s00432-022-04271-z

**Published:** 2022-09-07

**Authors:** Susanne Beyer, Lena Müller, Sophie Mitter, Lucia Keilmann, Sarah Meister, Christina Buschmann, Fabian Kraus, Nicole E. Topalov, Bastian Czogalla, Fabian Trillsch, Alexander Burges, Sven Mahner, Elisa Schmoeckel, Sanja Löb, Stefanie Corradini, Mirjana Kessler, Udo Jeschke, Thomas Kolben

**Affiliations:** 1 Department of Obstetrics and Gynecology, University Hospital, LMU Munich, Munich, Germany; 2 Institute of Pathology, University Hospital, LMU Munich, Munich, Germany; 3 Department of Gynecology and Obstetrics, University Hospital Wuerzburg, Würzburg, Germany; 4 Department of Radiation‑Oncology, University Hospital, LMU Munich, Munich, Germany; 5 Department of Obstetrics and Gynecology, University Hospital Augsburg, Augsburg, Germany

**Keywords:** RIG-I, DDX58, EFTUD 2, Endometrial cancer, Survival, Innate immune system

## Abstract

**Purpose:**

Endometrial cancer is the most common gynecological malignancy. The helicase RIG-I, a part of the innate immune system, and EFTUD2, a splicing factor which can upregulate RIG-I expression, are shown to influence tumor growth and disease progression in several malignancies. For endometrial cancer, an immunogenic cancer, data about RIG-I and EFTUD2 are still missing. The aim of this study was to examine the expression of RIG-I and EFTUD2 in endometrial cancer.

**Methods:**

225 specimen of endometrial cancer were immunohistochemically stained for RIG-I and EFTUD2. The results were correlated to clinicopathological data, overall survival (OS) and progression-free survival (PFS).

**Results:**

High RIG-I expression correlated with advanced tumor stages (FIGO: *p* = 0.027; pT: *p* = 0.010) and worse survival rates (OS: *p* = 0.009; PFS: *p* = 0.022). High EFTUD2 expression correlated to worse survival rates (OS: *p* = 0.026; PFS: *p *< 0.001) and was determined to be an independent marker for progression-free survival.

**Conclusion:**

Our data suggest that the expression of RIG-I and EFTUD2 correlates with survival data, which makes both a possible therapeutic target in the future.

**Supplementary Information:**

The online version contains supplementary material available at 10.1007/s00432-022-04271-z.

## Introduction

Endometrial carcinoma (EC) is the most common gynecologic malignancy and the sixth most common cancer among women worldwide (Bray et al. 2018) with an increasing incidence (Society. 2014). In 2018, an incidence of 382.069 cases and a mortality of almost 90.000 worldwide was estimated by *The International Agency for Research and Cancer* (Bray et al. 2018). Several risk factors are known, including obesity, diabetes mellitus, early menarche, nullipara or therapy with tamoxifen (Braun et al. 2016). It was thought that all those factors could contribute to an imbalance in the estrogen level which favors cell transformation. Previously, Bokhman differentiated between EC Type I (estrogen-dependent) and Type II (estrogen-independent) (Bokhman 1983). Today, EC is classified according to the ProMisE algorithm. This classification system includes MMR deficiency, POLE mutation, p53 wildtype and p53 aberrancy (Kommoss et al. 2018). The therapy for EC is multimodal and contains surgery, radiotherapy and chemotherapy (Concin et al. 2021). In Germany, a relative 10-year survival rate of 74% is estimated (RKI 2021). Nevertheless, about 20% are diagnosed in advanced stages (FIGO III-IV) and up to 12% suffer from recurrence (Huijgens and Mertens 2013, Hong et al. 2022). In these situations, the prognosis is worse with an estimated 5-year survival rate of just about 17% due to limited therapeutic options (Legge et al. 2020, Siegel et al. 2021). Therefore, new therapeutic strategies are needed and there is a rising interest in immunecheckpoint inhibitors. The PD-1-inhibitor Pembrolizumab showed promising results in clinical trials and in combination with the tyrosine kinase inhibitor lenvatinib even significantly better survival rates (Ott et al. 2017; Marabelle et al. 2020; Makker et al. 2022). Recent studies identified immune-related genes and proteins as well as the microenvironment of the tumor as relevant prognostic factors in EC (Chen et al. 2020; Ding et al. 2020). As many cancer cells develop strategies to escape the immune response, new insights into the understanding of this process could contribute to the development of new therapeutic options.

A major initiator of the innate immune response is the retinoic acid-inducible gene I, RIG-I, a cytosolic helicase. RIG-I is encoded by the gen DDX58 and together with MDA-5 and LGP-2, it belongs to the family of RIG-I-like receptors (Onoguchi et al. 2011) and detects viral single and double stranded RNA particles. By binding to viral RNA, a signaling process is initiated. This leads to an activation of the transcription factor NF-kB and finally to an upregulation of proinflammatory genes, including type I and III interferons and IFN stimulated genes (Quicke et al. 2017). In malignancies activation of innate immune signaling has been widely reported in absence of infections and plays and active role in the regulation of broader immune response. In ovarian cancer, RIG-I activation leads to an upregulation of MHC class I and to a secretion of proinflammatory mediators leading to the hypothesis that this could increase antitumor activity within microenvironment (Kübler et al. 2010). In HCC, a downregulation of RIG-I leads to enhanced levels of IFN alpha and was associated to poorer prognosis (Hou et al. 2014). It seems to be clear, that RIG-I has multiple roles in cancer tissue (Xu et al. 2018), including induction of intrinsic and extrinsic apoptosis (Elion et al. 2018; Castiello et al. 2019, Rameshbabu et al. 2021). RIG-I can be induced by many upstream factors, among them an induction by the Elongation factor Tu GTP-binding domain containing 2 (EFTUD2) is notable (Breiman et al. 2005; Stok et al. 2020).

EFTUD2 is a component of the spliceosome and an important protein regulating alternative splicing. EFTUD2 mutations are reported to cause mandibulofacial dysostosis with microcephaly (Lines et al. 2012), in which context it was originally detected. In the past years, it has been demonstrated that EFTUD2 is involved in various biological processes as it modulates cell differentiation, influences the innate immune response (Asghar and Meyer 2012; Zhu et al. 2015a, b) and the spliceosome activation (Guo et al. 2014).

To the best of our knowledge, this is the first study examining the protein expression of RIG-I and EFTUD2 on EC by immunohistochemistry.

## Materials and methods

### Patients and specimen

Native tissue samples were systematically accumulated between 1990 and 2001 from patients who underwent surgery at the Department of Gynecology and Obstetrics, Ludwig-Maximilians-University Munich, Germany. The surgically removed material (hysterectomy specimens and if indicated pelvic/paraaortic lymph nodes (for details about indication of lymphonodectomie see: (AGO-S3-Leitlinie: Diagnostik, T. u.N.d.P.m.E. 2018)) was immediately fixed in formaldehyde and embedded in paraffin for diagnosis and for further experiments.

In this study, we included a total number of 225 patients with endometrioid EC.

The average age at first diagnosis was 65 years, with the youngest patient being 35 and the oldest 85 years old. The average time of follow-up was about 11 years (131 months). In this context, we distinguished between overall survival (OS = time between diagnosis and all-cause death of the patient or last follow-up) and progression-free survival (PFS = time from diagnosis to any kind of progression, also including all-cause death).

Staging was performed based on the criteria formulated by the Fédération Internationale de Gynécologie et d’Obstétrique (FIGO) based on its version of 2020 (AGO-S3-Leitlinie), when all samples were re-staged by a pathologist according to the current staging system (Supplement 1). Grading types were assigned according to the WHO criteria by a gynecological pathologist, who also verified the histological subtype.

The majority of patients was diagnosed at an early stage of the disease (> 80% in stage I and II) and more than half of the tumor specimens presented a low-grade carcinoma (56% G1). Details concerning the study cohort are provided in (Table 1).

**Table 1:** Clinic pathological variables of the patients included in this study

Item	Patient no.(*n* = 225)	%
Age at diagnosis (years) ≤ 65 > 65	109 116	48.4 51.6
Tumor size (pT) pT1 pT2 pT3 pT4 Not available	175 16 30 3 1	77.8 6.4 13.3 1.3 0.4
FIGO staging I II III IV Not available	167 15 36 6 1	74.2 6.7 16.0 2.7 0.4
Grade G1 G2 G3 Not available	127 77 20 1	56.4 34.2 8.9 0.4
Nodal status (pN) pN0 pN1 pNX	142 21 62	63.1 9.3 27.6
Metastases (pM) pM0 pM1 pMX	109 5 111	48.4 2.2 49.3
Survival Alive Dead Not available	93 132 0	41.3 58.7 0.0
Progression		
None At least one Not available	180 45 0	80.0 20.0 0.0

pT: Tumor size (pT1: The cancer is found only in the uterus; pT2: The tumor has spread from the uterus to the cervical stroma but not to other parts of the body; pT3: The cancer has spread beyond the uterus, but it is still only in the pelvic area; pT4: The cancer has spread to the mucosa of the rectum or bladder). FIGO: classification of the International Federation of Gynecology and Obstetrics (FIGO I: The cancer is found only in the uterus; FIGO II: The tumor has spread from the uterus to the cervical stroma but not to other parts of the body; FIGO III: The cancer has spread beyond the uterus, but it is still only in the pelvic area; FIGO IV: The cancer has spread to the mucosa of the rectum or bladder or the cancer has spread to lymph nodes in the groin area, and/or it has spread to distant organs, such as the bones or lungs). pN: status regarding lymph node. pN0: histopathological lymphnodes without cancer infiltration. pN1: histopathological lymphnodes with cancer infiltration. pNX: no information about lymph nodes in the histopathological examination, due to missing lymph node sampling. pM: status regarding distant metastasis. pM0: histopathological examination without sign for distant metastasis. pM1: histopathological examination with sign for distant metastasis. pMX: no information about distant metastasis in the histopathological examination

The patient and tumor characteristics as well as the follow-up data were provided by the Munich cancer registry. The data are completely anonymized.

### Methods

#### Immunohistochemistry of RIG-I and EFTUD2

The EC samples were fixed in neutral-buffered formalin and underwent paraffin embedding following a standard protocol. To perform the following examinations at a more standardized level and for a better comparison, tissue microarrays (TMAs) were established. For this purpose, the tumor was marked in the paraffin block and three columnar biopsies were taken from each of it. Those tumor columns were cast again with paraffin into blocks, now containing tissue samples of several patients at ones, which made the staining process more effective, and the scoring less error-prone due to randomized selection of visual fields within the tumor. The blocks were cut into 3 µm thick slices using a microtome (Hn40 Schlitten-Mikrotom, then Reichert-Jung, today part of Leitz, Wetzlar, Germany) and mounted on microscope slides. After deparaffinisation in Roticlear^®^ (Carl Roth, Arlesheim, Switzerland) and rinsing in ethanol, the tissue sections were blocked with 3% H_2_O_2_ in methanol at room temperature for 20 min to inactivate the endogenous peroxidase and subsequently rehydrated in a descending ethanol gradient. Afterwards, the slides were cooked in a pressure cooker using either an EDTA-based buffer containing surfactant (for RIG-I-staining) or a trisodium citrate buffer solution with pH = 6 (for EFTUD2 staining) aiming for epitope retrieval. Blocking solution was applied for blocking of the non-specific binding of the primary antibody. Tissue sections were incubated with the primary antibody. Subsequently, substrate and chromogen were added to the slides. For detailed staining process, see Table 2. After counterstaining with Mayer’s acidic haematoxylin (Waldeck, Münster, Germany), the slides passed through an ascending alcohol series and were finally cover slipped. Appropriate negative and positive controls were included in the staining (Supplement 2).

**Table 2:** Overview of antibodies and chemicals used in the staining process

RIG-I	EFTUD2
Blocking solution^a^: 20 min	Blocking solution^b^: 5 min
Primary antibody^c^	Primary antibody^d^
1:100 in AD^e^	1:300 in PBS^f^
Incubation 1 h; 21 °C	Incubation 16,5 h; 4 °C
ImmPRESS polymer reagent^g^: 30 min	PostBlock^h^: 20 min
	HRP Polymer^i^: 30 min
Chromogen: DAB^j^, 3 min	Chromogen: DAB^j^, 2 min

^a^Normal Horse Serum (NHS), 2.5%, ImmPRESS™ Anti-Mouse IgG Polymer Kit, VECTOR laboratories, Newark, United States; catalogue number: MP-7402

^b^ZytoChem Plus HRP Polymer Kit (Mouse/Rabbit) 3 × 100, Zytomed Systems GmbH, Berlin, Germany; catalogue number POLHRP-100

^c^RIG-I-antibody: monoclonal IgG (mouse); LSBio, Seattle, United States; catalogue number: LS-C331000

^d^EFTUD2-antibody: polyclonal IgG (rabbit); Abcam, Cambridge, United Kingdom; catalogue number: ab72456

^e^Antibody Diluent, Agilent Technologies, Santa Clara, United States; catalogue number: S3022

^f^Dulbecco ‘s Phosphate Buffered Saline, SigmaAlderich, Saint Louis, Missouri, United States

^g^Anti-Mouse IgG Polymer Kit, VECTOR laboratories, Newark, United States; catalogue number: MP-7402

^h^ZytoChem Plus HRP Polymer Kit (Mouse/Rabbit); Zytomed Systems GmbH, Berlin, Germany

^i^Vectastain Elite ABC Kit, diluted NORMAL serum; Vector laboratories, Newark, United States

^j^Liquid DAB + Substrate Chromogen System, REF K3468; Abcam, Cambridge, United Kingdom

To evaluate the staining, the immunoreactive score (IRS) was used (Remmele et al. 1986). By this semi-quantitative score, the intensity of the staining (0 = not stained; 1 = low intensity; 2 = moderate intensity; 3 = high intensity) and the percentage of stained cells (0 = 0%; 1 = 1–10%; 2 = 11–50%; 3 = 51–80%; 4 > 80%) was multiplied, resulting in a number between 0 (weak expression) and 12 (strong expression). Since we evaluated three different tumor areas from each patient, this also resulted in three IRS values, whereas we calculated the average.

### Statistical analysis

The statistical analysis was carried out by Microsoft Excel and SPSS Statistics 26 (SPSS Inc., Chicago, Illinois). Comparisons of independent groups were conducted using non-parametric tests for two (Wilcoxon–Mann–Whitney *U* test) or multiple groups (Kruskal–Wallis test). All histopathological variables as provided in the table above and the expression of RIG-I/EFTUD2 were tested for correlations using Spearman’s Rho. Survival times were compared by Kaplan–Meier analysis, and differences in OS and PFS of patients were tested for significance by the log-rank test. A multivariate Cox regression model was established for the analysis of survival to compare the risk of death in patients depending on the expression. In addition to the expression levels, the effects of further independent factors such as age at diagnosis, tumor grade, tumor size, lymph node status and FIGO classification were accounted for in this model. The one in ten rule (Vittinghoff and McCulloch 2007) was respected and only variables with a complete dataset were included. *P* values were two sided and had to be lower than *p* < 0.05 for being considered to be statistically significant.

### Ethics approval

The study was approved by the local ethics committee of the Ludwig-Maximilians-University of Munich (reference number 19-249, 2019) and was performed in accordance to the Declaration of Helsinki.

## Results

### pT, FIGO and grade correlate to RIG-I expression

**Fig. 1:**
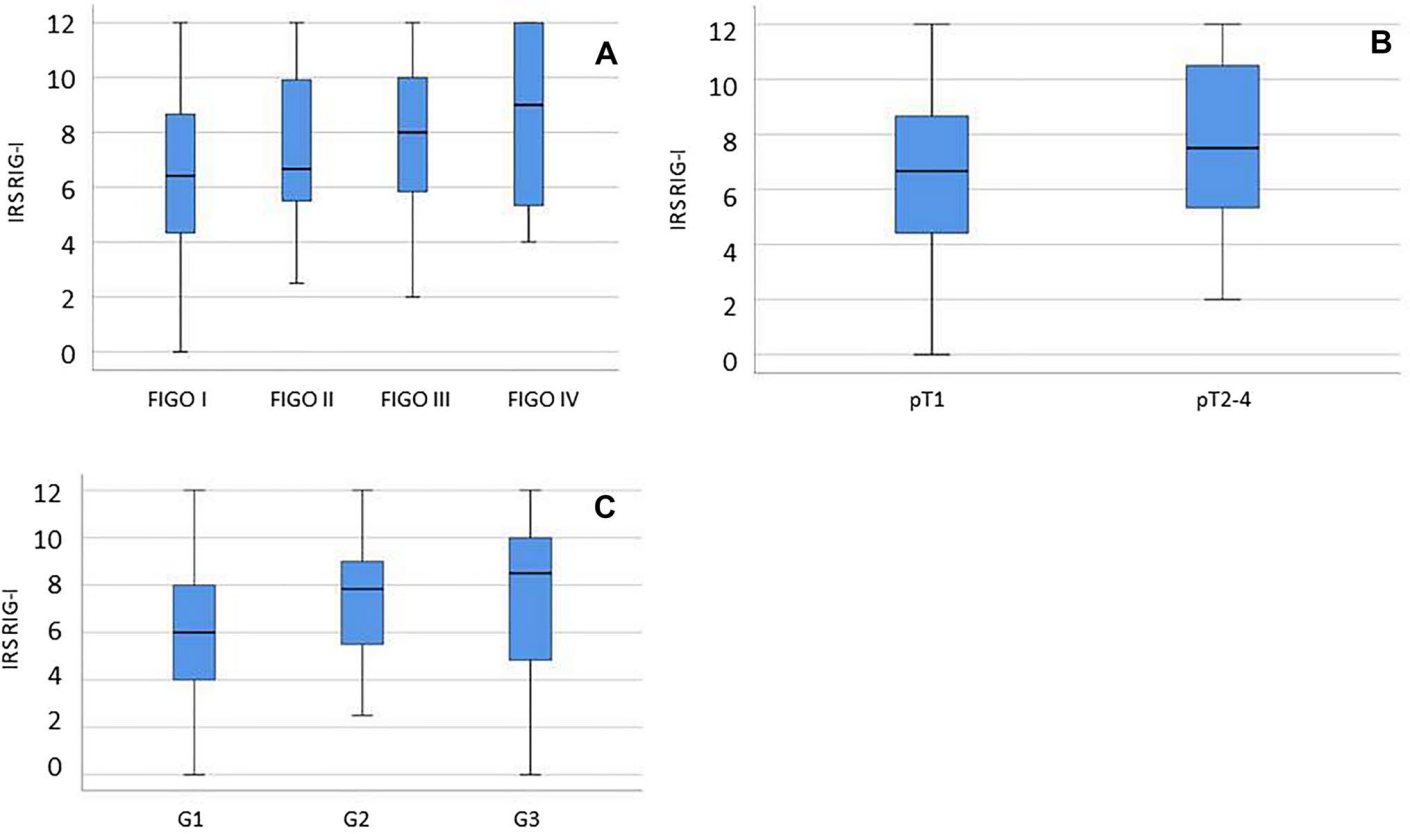
RIG-I expression and clinicopathological variables. RIG-I expression is significant lower in FIGO I (tumor is found only in the uterus) compared to FIGO IV (tumor has spread to the mucosa of rectum/bladder or to lymph nodes in the groin area or to distant organs) as shown in the boxplot (**A**). Regarding pT (tumor size) stages, RIG-I expression is lower in pT1 (tumor is only found in the uterus) compared to higher pT stages (**B**). Low graded endometrial cancers have significant less RIG-I expression than high graded samples, see in boxplot (**C**)

99% of all EC specimen expressed RIG-I in the cytoplasm. The median IRS was 7.0 (5.4%). RIG-I was found to be expressed in the nucleus only very weakly for which reason the following results refer to cytoplasmic RIG-I expression. A low cytoplasmic expression (IRS ≤ 4) was detected in 21.2% compared to a high expression (IRS > 4) in 78.8% of all cases. In the further course, we correlated clinical-pathological data with the IRS of RIG-I (Table 3). RIG-I expression was significantly increased in higher FIGO stages (*p* = 0.027, Fig. 1A; Supplementary 3). Furthermore, RIG-I levels correlated with the size of the primary tumor (pT stage): patients whose cancer was diagnosed early as a pT1 tumor showed significantly lower RIG-I expression (*p* = 0.010, Fig. 1B; Supplementary 3). The higher the tumor grade the patients presented, the higher the IRS we determined on the corresponding histological specimens (*p* = 0.007; Fig. 1C; Supplementary 3). There was no significant correlation with RIG-I in terms of lymph node (pN) and distant metastases (pM).

**Table 3:** Staining results and correlation analysis

	RIG-I				EFTUD2			
	Median IRS (± SD)	%	*p* (NPAR)	*ρ*	Median IRS (± SD)	%	*p* (NPAR)	*ρ*
FIGO^1^								
FIGO I FIGO II FIGO III FIGO IV	6.4 (± 2.6) 6.7 (± 2.9) 8.0 (± 2.7) 9.0 (± 3.4)	/* 6.7 11.1 0	**0.027**	0.201	6.0 (± 3.1) 4.3 (± 2.7) 5.8 (± 3.4) 6.3 (± 3.2)	7.5 6.7 /* 0.0	0.815	− 0.044
pT^2^								
T1 T2/3/4	6.7 (± 2.6) 7.5 (± 3.0)	2.3 2.0	**0.010**	0.174	6.00 (± 3.1) 5.7 (± 3.1)	7.7 8.2	0.928	0.006
Grade^1^								
G1 G2 G3	6.0 (± 2.6) 7.8 (± 2.4) 8.5 (± 3.7)	10.3 /* 0	**0.007**	0.210	5.7 (± 3.1) 6.0 (± 3.2) 6.8 (± 3.5)	3.2 10.7 /*	0.303	0.096
pN^2^								
N − N +	6.7 (± 2.7) 8.0 (± 2.9)	1.4 19.0	0.753	0.110	6.0 (± 3.1) 5.7 (± 3.5)	8.0 4.8	0.657	− 0.035
pM^2^								
M − M +	7.0 (± 2.5) 5.3 (± 4.5)	6.5 20.0	0.539	0.025	5.5 (± 3.1) 6.7 (± 4.3)	/* 20.0	0.464	0.102

*SD* standard deviation, *%* percentage of the subgroup with median IR, *NPAR * non-parametric test, *p*  p value, *ρ*  correlation coefficient. ^1^correlation tested by Kruskall–Wallis test (p; NPAR) and Spearman-correlation (ρ).^2^correlation tested by Mann–Whitney test (p; NPAR) and Spearman-correlation (ρ).

FIGO: classification of the International Federation of Gynecology and Obstetrics (FIGO I: The cancer is found only in the uterus; FIGO II: The tumor has spread from the uterus to the cervical stroma but not to other parts of the body; FIGO III: The cancer has spread beyond the uterus, but it is still only in the pelvic area; FIGO IV: The cancer has spread to the mucosa of the rectum or bladder or the cancer has spread to lymph nodes in the groin area, and/or it has spread to distant organs, such as the bones or lungs). pT: Tumor size (pT1: The cancer is found only in the uterus; pT2: The tumor has spread from the uterus to the cervical stroma but not to other parts of the body; pT3: The cancer has spread beyond the uterus, but it is still only in the pelvic area; pT4: The cancer has spread to the mucosa of the rectum or bladder). pN: status regarding lymph node. pN0: histopathological lymphnodes without cancer infiltration. pN1: histopathological lymphnodes with cancer infiltration. pM: status regarding distant metastasis. pM0: histopathological examination without sign for distant metastasis. pM1: histopathological examination with sign for distant metastasis. *Values resulted from calculations, no sample with this value exists. Significant results are shown in bold

### EFTUD2 does not correlate to clinicopathological variables

**Fig. 2:**
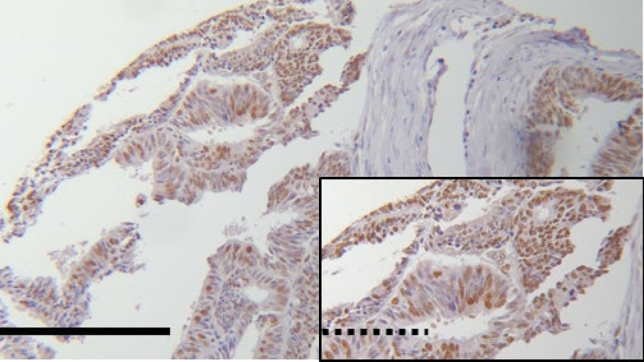
Exemplary image of endometrial cancer tissue with high (IRS = 8) EFTUD2 expression (B). Magnification: big pictures *25, small pictures *100

Due to technical issues (specimen floating off the slides during the staining process), we could only use 219 samples in our analysis of EFTUD2 expression. Out of these 219 samples, none or very low EFTUD2 expression (IRS ≤ 1) was displayed in 5.5% of the specimens, whereas an IRS ≥ 9 was shown in 51 (23.3%) cases. The median IRS of EFTUD2 staining was 6.00. 55.3% of all samples showed an EFTUD2 expression ≤ 6, whereas 44.7% scored higher than the median IRS (example see Fig. 2). Examining the EFTUD2 expression with regards to tumor size, pN-/pM-status, grade and FIGO classification, we could not find statistically significant differences and there was no significant correlation between IRS and any of the clinicopathological parameters mentioned above (Table 3).

### High RIG-I level is correlated with poor survival in EC

**Fig. 3:**
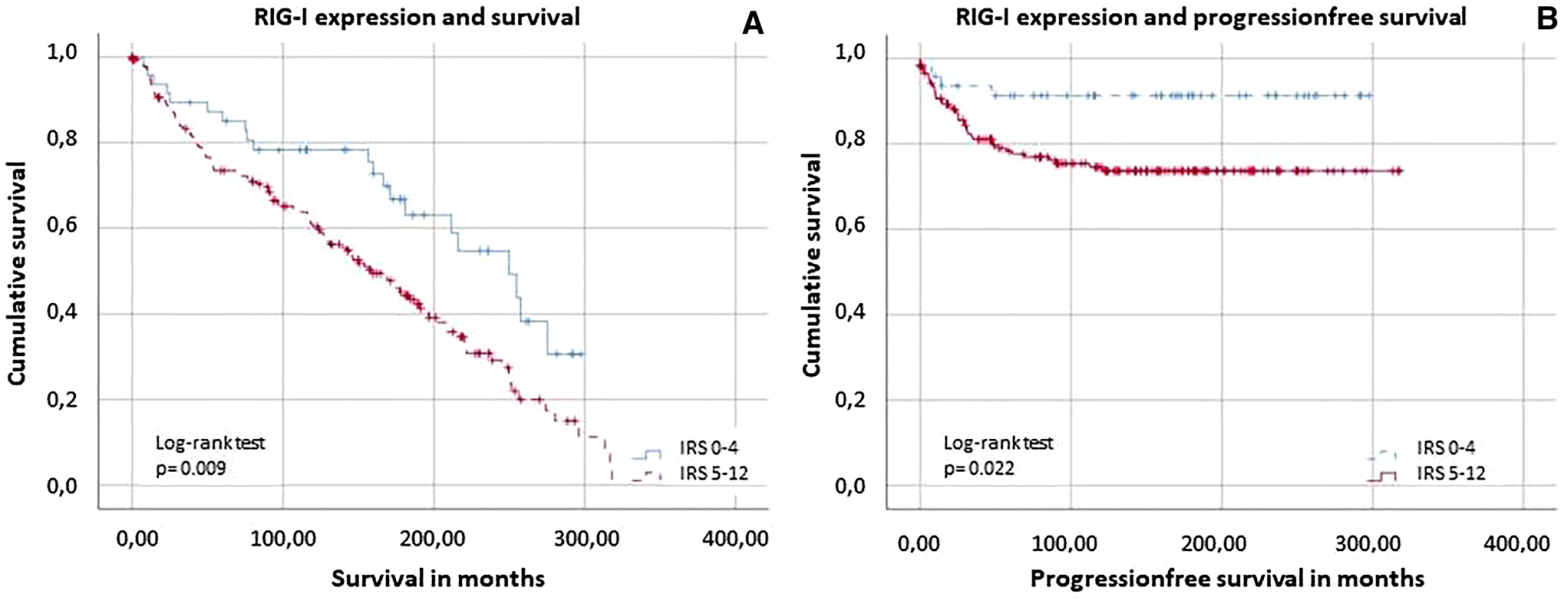
Survival rates depending of RIG-I expression. Patients with low RIG-I expression (IRS ≤ 4) have significant better overall survival- (*p* = 0.009; **A**) and progression-free survival- (*p* = 0.022; **B**) rates (tested by log-rank test). For distribution of the patient group, see Supplement 4

As shown in the data above, RIG-I expression correlates at a significant level with various clinicopathological data. Additionally, our data strongly suggest that RIG-I levels in endometrioid adenocarcinoma are associated with both PFS and OS. With a cut-off at IRS = 4, univariate time-to-event analyses using the Kaplan–Meier estimator showed a significantly reduced OS in patients with high RIG-I expression (*p* = 0.009, Fig. 3A). Patients with low RIG-I levels survived 207 months on average, whereas patients with a high IRS score only survived 159 months after diagnosis (95% confidence interval of the median: 197–303 vs. 129–189 months; for distribution of the groups see Supplement 4). Furthermore, high RIG-I levels were also associated with poor PFS in endometrioid adenocarcinoma patients (*p* = 0.022, Fig. 3B). In the group of patients with low expression (IRS ≤ 4), 8.5% of the study participants experienced progression within the observation period. This contrasts with 23.4% of patients suffering progression who presented with high RIG-I levels (IRS > 4). To assess whether RIG-I expression was an independent predictor for survival, we performed a multivariate Cox regression analysis. Neither OS nor PFS showed independent prognostic significance (Tables 4, 5). For correlation in between the histopathological variables and the categorization of the variables, see Supplement 5 and 6.

**Table 4:** Multivariate COX regression of RIG-I regarding overall survival

	Significance	HR	L95%	U95%
RIG-I expression	0.733	1.092	0.658	1.813
Age at diagnosis	**0.000**	1.062	1.039	1.086
Grade	0.137	1.315	0.917	1.887
pT	0.056	4.009	0.966	17.403
pN	**0.000**	2.462	1.683	3.601
FIGO	0.324	0.488	0.117	2.030

*HR* hazard ratio, *L95%* Lower 95% CI of Exp(B), *U95%* upper 95% CI of Exp(B), *pT* tumor size, *pN* status regarding lymph node. FIGO: classification of the International Federation of Gynecology and Obstetrics. For categorization of variables, see Supplement 7. Significant results are shown in bold

**Table 5:** Multivariate COX regression of RIG-I regarding progression-free survival

	Significance	HR	L95%	U95%
RIG-I expression	0.173	2.100	0.722	6.104
Age at diagnosis	0.901	0.998	0.967	1.030
Grade	**0.040**	1.926	1.032	3.596
pT	0.291	1.970	0.560	6.935
pN	**0.019**	2.184	1.137	4.195
FIGO	0.459	1.634	0.446	5.987

*HR *hazard ratio, *L95%*  Lower 95% CI of Exp(B), *U95%* upper 95% CI of Exp(B), *pT* tumor size, *pN* status regarding lymph node. FIGO: classification of the International Federation of Gynecology and Obstetrics. For categorization of variables, see Supplement 7. Significant results are shown in bold

### High EFTUD2 level is correlated with poor survival in endometrial cancer

**Fig. 4:**
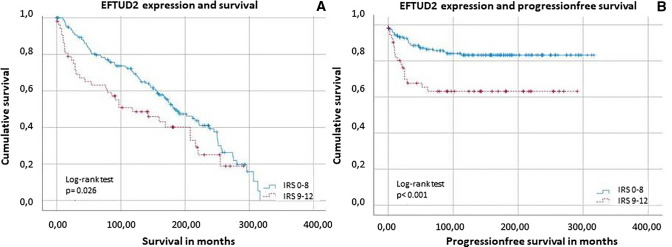
Kaplan–Meier analysis for overall survival **A** and progression-free survival **B** with regards to EFTUD2 expression with significant better survival rates when EFTUD2 expression was IRS 0–8 (tested by log-rank test). For distribution of the patient group, see Supplement 5

Although EFTUD2 expression was not associated with tumor stage, its expression correlated with OS and PFS. As shown in Fig. 4, patients with low EFTUD2 expression (IRS ≤ 8) had significantly (*p* = 0.026/*p* < 0.001) better OS and PFS than patients with higher EFTUD2 expression. The analysis also showed that the median survival time of patients with high EFTUD2 (IRS > 8) expression was only 118 moths (95% confidence interval of the median: 150–218 months) and an event of progression occurred in 33.96% of cases. In comparison, patients with lower (IRS ≤ 8) EFTUD2 expression showed a median survival time of 184 moths (95% confidence interval: 39–198 months) and only progressed in 15.06% of cases.

EFTUD2 expression turned out to be an independent marker for OS and PFS in the multivariate Cox regression (Tables 6, 7). For the distribution of the groups, see Supplement 7.

**Table 6:** Multivariate COX regression of EFTUD2 regarding overall survival

	Significance	HR	L95%	U95%
EFTUD2	0.064	1.474	0.978	2.221
Age at diagnosis	**0.000**	1.059	1.037	1.081
Grade	0.215	1.263	0.873	1.827
pT	0.051	4.234	0.996	18.004
pN	**0.000**	2.669	1.811	3.934
FIGO	0.311	0.479	0.115	1.989

Significant results are shown in bold

*HR* hazard ratio, *L95%* lower 95% CI of Exp(B), *U95%* upper 95% CI of Exp(B), *pT* tumor size, *pN* status regarding lymph node. FIGO: classification of the International Federation of Gynecology and Obstetrics. For categorization of variables, see Supplement 7

**Table 7:** Multivariate COX regression of EFTUD2 regarding progression-free survival

	Significance	HR	L95%	U95%
EFTUD2	**0.005**	2.497	1.326	4.701
Age at diagnosis	0.783	0.996	0.965	1.027
Grade	0.152	1.603	0.841	3.055
pT	0.353	1.824	0.512	6.494
pN	**0.017**	2.236	1.152	4.338
FIGO	0.258	2.109	0.578	7.686

Significant results are shown in bold

*HR *hazard ratio, *L95%*  Lower 95% CI of Exp(B), *U95%* upper 95% CI of Exp(B), *pT* Tumor size, *pN* status regarding lymph node. FIGO: classification of the International Federation of Gynecology and Obstetrics. For categorization of variables, see Supplement 7

## Discussion

RIG-I is expressed in most human cells and plays an important role in the innate immune system (Horvath et al. 2020). As already revealed in functional experiments, its expression can be upregulated by the splicing factor EFTUD2 (Zhu et al. 2015a, b). Although EC is known as an “immunogenic” cancer, this is—to the best of our knowledge—the first study examining RIG-I and EFTUD2 in EC. We detected that a high expression of RIG-I correlated with high FIGO and pT stages as well as higher grade. Additionally, high expression of RIG-I and EFTUD2 was associated with poor PFS.

RIG-I is a nucleoid acid receptor in the cytoplasm (Wicherska-Pawlowska et al. 2021). In addition, recent studies showed that RIG-I can be located in the nucleus in some cells (Liu et al. 2018). The ligands of RIG-I (various RNA molecules) are typically (but not exclusively) parts of the viral RNA (Gack et al. 2007). After ligand biding, RIG-I interacts with the MAVS (Breiman et al. 2005; Matsumiya and Stafforini 2010). This process results in the activation of NFĸB and IRF-3 and leads to the production of Type I IFN, chemokines and other cytokines (Seth et al. 2005). By these substances, effector *T* cells and NK cells are stimulated leading to a tumor-suppressive effect (Elion and Cook 2018). RIG-I can also activate intrinsic and extrinsic apoptosis pathways (Rameshbabu et al. 2021). Taking these effects and pathways together, RIG-I as part of the innate immune system has become more and more important over the last years.

In several tumors, RIG-I activation follows the described mechanisms and leads to a tumor-suppressive milieu. Well-studied examples are pancreatic cancer and melanoma (Poeck et al. 2008; Ellermeier et al. 2013). RIG-I stimulation by endogenous RNAs enhanced therapy resistance by a STAT-1-dependent pathway and NOTCH signaling (Boelens et al. 2014). In breast cancer, a high RIG-I expression was also described to be associated with poor outcome: RIG-I stimulation by endogenous RNA lead to increased tumor growth and metastasis in vitro (Nabet et al. 2017).

This trend also appeared in our results as well as in ovarian cancer. In a retrospective study with 141 cases of ovarian cancer, RIG-I was overexpressed (compared to healthy ovarian tissue) and a high expression correlated with higher tumor grade as well as to poorer outcome (Wolf et al. 2020). Additionally, correlations with interferon beta, PDL-1 and FOXP3 levels were detected in ovarian cancer (Wolf et al. 2020). The immune-suppressive system of PD-L1 was found to be associated with poor survival rates in ovarian cancer as well as in EC (Wieser et al. 2018; Zhang et al. 2021). The authors suggest that in ovarian cancer the antiviral and tumor-suppressive effects of RIG-I seem to be neutralized by an immune-escape phenomenon in the tumor microenvironment (Wolf et al. 2020). Regarding the tumor microenvironment in EC, it is known that regulatory *T* cells and their marker FOXP3 are correlated with worse survival rates (de Jong et al. 2009; Xi et al. 2019). By interaction with the tumor microenvironment RIG-I seems to lose its tumor-suppressive effect in ovarian cancer (Wolf et al. 2020). Discovery of mechanism how this protective function has been aborted could help to develop strategies for more effective use of immunotherapies in both EC and OC.

Still, it is important to keep in mind, that our results refer to the expression of RIG-I and not to its activation, similar to the study in ovarian cancer (Wolf et al. 2020). Nevertheless, RIG-I remains a potential target in further therapies and agonists as well as antagonists are in development (Rawling et al. 2020; Onomoto et al. 2021).

As an upregulation of RIG-I by EFTUD2 was described (Zhu et al. 2015a, b), we also investigated the expression of this splicing factor. EFTUD2 seems to be involved in viral infections and miscarriages (Zhu et al. 2015a, b; Löb et al. 2020). Only limited data are available examining the role of EFTUD2 in cancer and, to the best of our knowledge, this is the first study examining EFTUD2 in EC. We observed a significant correlation between EFTUD2 and survival rates in EC: a high expression was associated with low PFS and OS and turned out to be an independent marker for PFS. EFTUD2 is known to be a modulator of the innate immune system: in colorectal cancer, EFTUD2 seems to promote tumor growth, especially in a colitis-associated environment and by modulation of macrophages (Lv et al. 2019). Lv et al. demonstrated in a mouse model of colorectal cancer that a knockdown of EFTUD2 resulted in a reduced secretion of proinflammatory cytokines and tumorigenic factors (Lv et al. 2019). This reduction of inflammation and tumor development was related to an impaired activation of TLR-4-NF-κB signaling cascade in macrophages due to an altered EFTUD2 expression (Lv et al. 2019). In hepatic cellular cancer, EFTUD2 was upregulated compared to healthy liver tissue (Lv et al. 2021). In analogy to our results in EC, a negative correlation between survival rates and EFTUD2 expression was shown. Also, a reduced tumor growth in case of EFTUD2 knockdown was detected, suggesting it as an independent prognostic factor for patients with hepatic cellular cancer (Tu et al. 2020; Lv et al. 2021).

Furthermore, another limitation of this study needs to be discussed: although the data regarding FIGO/pT/grade and survival rates are consistent and clear, RIG-I expression did not turn out to be an independent marker for survival. This assumes that underlying mechanisms which are not fully understood play an important role. Further studies are, therefore, necessary to understand these processes.

In summary, our data show that a high RIG-I expression is correlated with advanced tumor stages and thus subsequently to worse survival rates. EFTUD2 turned out to be an independent marker for progression-free survival. Therefore, RIG-I and EFTUD2 seem to be negative predictors in patients with EC. To examine the underlying mechanisms, further studies are necessary.

## Supplementary Information

Below is the link to the electronic supplementary material.

Supplementary file1 (DOCX 3496 KB)

## Data Availability

All data generated or analyzed during this study are included in this published article. For any questions, please contact S. Beyer.

## Abbreviations

EC: Endometrial cancer
EFTUD2: Elongation factor Tu GTP-binding domain containing 2
OS: Overall survival
PFS: Progression-free survival
RIG-I: Retinoic acid-inducible gene I
