# Supplementary material for: High RIG-I and EFTUD2 expression predicts poor survival in endometrial cancer
Source: J Cancer Res Clin Oncol. 2022 Sep 7;149(8):4293–303. doi: 10.1007/s00432-022-04271-z (PMC10349715; doi:10.1007/s00432-022-04271-z)
Supplement: Supplementary file 1 — Supplementary file1 (DOCX 3496 KB) [file 432_2022_4271_MOESM1_ESM.docx]

**Supplements**

**Supplement 1.** FIGO stages of endometrial cancer.

| TNM category | FIGO stage | Definition |
| --- | --- | --- |
| T1 | I | The cancer is found only in the uterus |
| T1a | IA | The cancer is found only in the endometrium or less than one-half of the myometrium. |
| T1b | IB | The tumor has spread to one-half or more of the myometrium. |
| T2 | II | The tumor has spread from the uterus to the cervical stroma but not to other parts of the body. |
| T3 and/or N1 | III | The cancer has spread beyond the uterus, but it is still only in the pelvic area. |
| T3a | IIIA | The cancer has spread to the serosa of the uterus and/or the tissue of the fallopian tubes and ovaries but not to other parts of the body. |
| T3b | IIIB | The tumor has spread to the vagina or to the tissue immediately next to the uterus called the parametrium. |
| N1 | IIIC | The cancer has spread to the regional pelvic or para-aortic lymph nodes |
| T4 | IVA | The cancer has spread to the mucosa of the rectum or bladder. |
| M1 | IVB | The cancer has spread to lymph nodes in the groin area, and/or it has spread to distant organs, such as the bones or lungs. |

**Supplement 2.** Negative (A) and positive (B) control for RIG-I and EFTUD2 staining (C: negative; D: positive control).

**
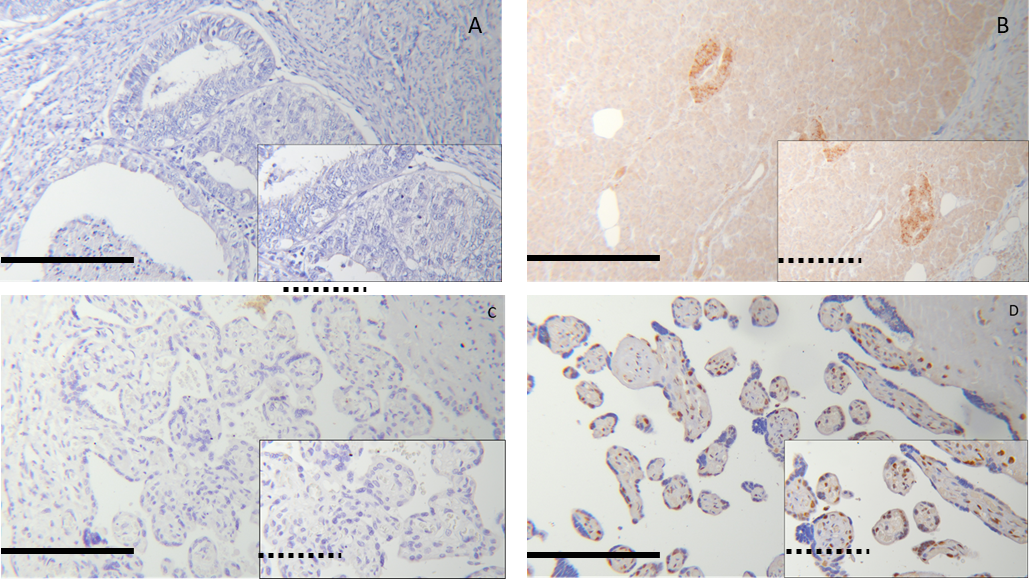
**

Magnification: big pictures *25, small pictures *100. = 200µm; =100µm

**Supplement 3.** RIG-I expression in correlation to clinicopathological variables.


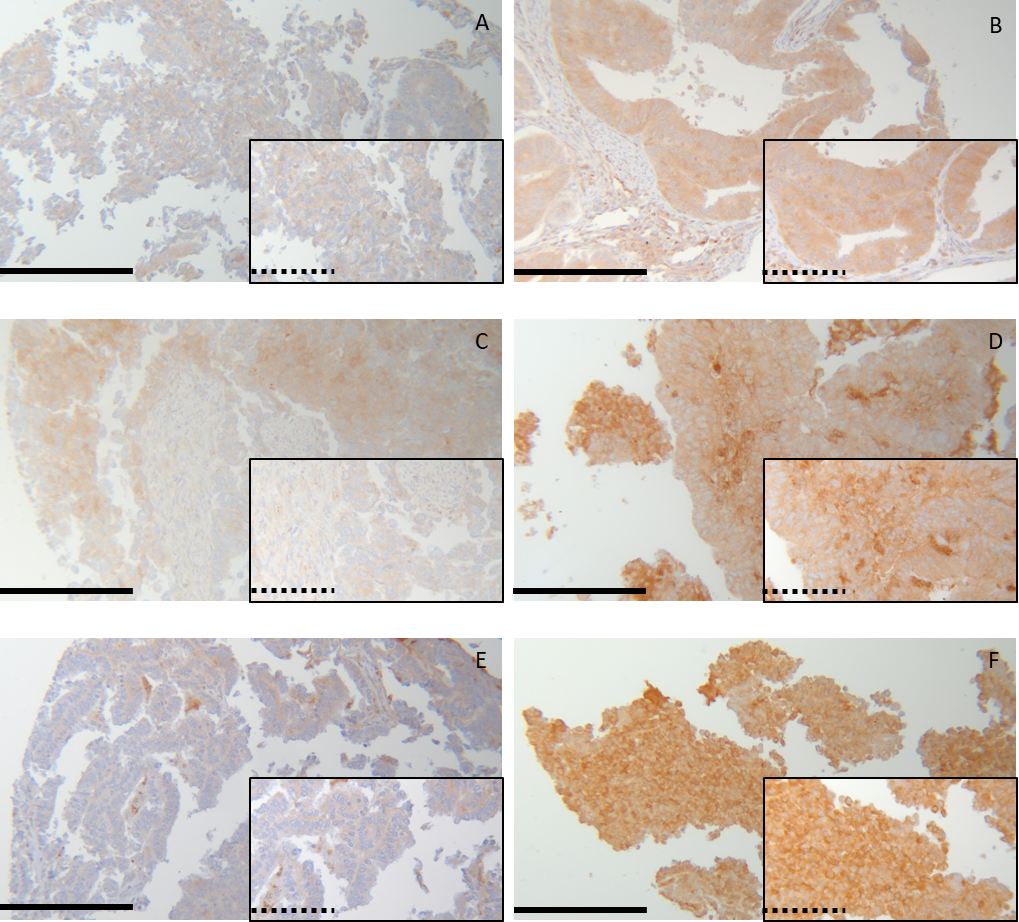


RIG-I Expression and clinicopathological variables. RIG-I expression is significant lower in FIGO I (tumor only found in the uterus; **A**) compared to FIGO IV (tumor has spread to the mucosa of rectum/bladder or to lymph nodes in the groin area or to distant organs; **B**). Regarding pT stages (tumor size), RIG-I expression is lower in pT1 (tumor is found only in the uterus; **C**) compared to higher pT stages (**D**). Low graded endometrial cancers have significant less RIG-I expression (**E**) than high graded samples (G3; **F**). Magnification: big pictures *25, small pictures *100.
 = 200µm; =100µm

**Supplement 4**. Distribution of patient groups in survival analyses regarding RIG-I.

|  | **Total number** | **Number of events** | **Censored** |
| --- | --- | --- | --- |
| **Overall survival** |  |  |  |
| **IRS ≤4** | 47 | 21 | 26 |
| **IRS >4** | 175 | 109 | 66 |
| **Progression-free survival** |  |  |  |
| **IRS ≤4** | 47 | 4 | 43 |
| **IRS >4** | 175 | 41 | 134 |

**Supplement 5.** Correlation in between histopathological variables (performed by Kruskal-Wallis test).

|  | **pN** | **FIGO** | **Grade** | **Age** |
| --- | --- | --- | --- | --- |
| **pT** | 0.001 | <0.001 | <0.001 | 0.889 |
| **pN** | - | <0.001 | 0.412 | <0.001 |
| **FIGO** | - | - | <0.001 | 0.535 |
| **Grade** | - | - | - | 0.874 |

**Supplement 6.** Categorization of variables included in the multivariate Cox regression

| RIG-I | IRS 0-4 versus IRS 5-12 |
| --- | --- |
| EFTUD2 | IRS 0-8 versus IRS 9-12 |
| Age at diagnosis | continously |
| Grade | G1 versus G2 /G3 |
| pT | pT1 versus pT2/3/4 |
| pN | pN0 versus not pN0 |
| FIGO | FIGO I versus FIGO II-IV |

**Supplement 7.** Distribution of patient groups in survival analyses regarding EFTUD2.

|  | **Total number** | **Number of events** | **Censored** |
| --- | --- | --- | --- |
| **Overall survival** |  |  |  |
| **IRS ≤8** | 172 | 96 | 76 |
| **IRS >8** | 56 | 36 | 20 |
| **Progression-free survival** |  |  |  |
| **IRS ≤8** | 172 | 28 | 144 |
| **IRS >8** | 56 | 19 | 37 |
